# J. H. Logan, M. D.

**Published:** 1885-05

**Authors:** 


### J. H. LOGAN, M. D.

As already announced in our last number, the death of Dr John H. Logan occurred in this city on the 29th of March. After a protracted illness, during which his colleagues rendered most grateful attendance, and his numerous friends their warmest sympathy, he sank under pulmonary disease.

Dr. Logan was born November 5th, 1820, in Abbeville, S. C., and grew up amidst the best associations, imparting that elevation of sentiments which marked his career in life. He graduated with distinction for his high literary attainments in 1844, at Columbia, S. C., and received his medical degree in 1847 at Charleston. Both of these institutions enjoyed great celebrity at home and abroad, so that his State pride was gratified by graduating in his native State in the literary and medical departments.

He practiced medicine for several years near Ninety-Six, in Abbeville district, but was made principal of the high school at Abbeville in 1851, and retired from the duties of his profession for two years. Thence he went to Greenwood, in the same district, the former home of Mrs. Logan, a daughter of Dr. Calhoun, of that place. The practice of medicine was resumed then and continued until the opening of our civil war.

As a private in Capt. John Leland's company, he went forth in the discharge of duty with unflinching firmness, but was eventually assigned more appropriate service in the medical department. After being on duty for sometime at Winder Hospital in Richmond, Va., he served as surgeon with the 7th Regiment S. C. V., through the arduous campaigns in Virginia and Pennsylvania.

At the close of the war, Dr. Logan located at Silver Run, in Talladega county, Alabama, for the practice of medicine. But his recognized literary attainments and capacity of a high order for the responsible work of instruction caused a demand very soon for his superintendence of the Synodical Female College at Talladega. He continued in charge of that institution until elected Professor of Chemistry in Oglethorpe College at Atlanta, and upon its dissolution he engaged in teaching a private school in this city. With his ripe scholarship and taste for books, teaching was more congenial employment for him than the drudgery of professional life, but he was destined to combine these duties in an acceptable field of service. Dr. Logan was called to the chair of Chemistry in the Atlanta Medical College, and continued to discharge its duties in connection with the practice of medicine until disabled by sickness early in the present year. He manifested his sense of responsibility in the faithful discharge of all his obligations by preparing the interrogatories for the written examination of this graduating class. While confined to his room, and even when prostrated in bed, he examined their papers and signed their diplomas. Few men have struggled more heroically with the embarrassments of their situation, or manifested more energy in the performance of duty than this pure and noble man. A high-toned conviction of right and justice actuated his conduct throughout life, and his best efforts were employed for the promotion of good and useful objects among his fellow-men.

Dr. Logan was for a brief period editor of the *Atlanta Medical Register*. Among other literary productions, he wrote a history of South Carolina, and he made frequent contributions to the papers of the day, showing much practical discernment and great philanthropic devotion to the best interests of the community in which his lot was cast. He was an elder in the Presbyterian Church, and his pious life was closed with Christian faith and hope for the reward of the just in a future world of peace.

